# Exogenous glucose oxidation during endurance exercise under low energy availability

**DOI:** 10.1371/journal.pone.0276002

**Published:** 2022-10-12

**Authors:** Chihiro Kojima, Aya Ishibashi, Kumiko Ebi, Kazushige Goto

**Affiliations:** 1 Japan Institute of Sports Sciences, Nishigaoka, Kitaku, Tokyo, Japan; 2 Department of Life Science, Graduate School of Arts and Sciences, The University of Tokyo, Tokyo, Japan; 3 Graduate School of Sport and Health Science, Ritsumeikan University, Kusatsu, Shiga, Japan; Sport Sciences School of Rio Maior - Politechnic Institute of Santarem, PORTUGAL

## Abstract

The present study was conducted to determine the effect of endurance exercise under low energy availability (EA) on exogenous glucose oxidation during endurance exercise. Ten active males (21.4 ± 0.6 years, 170.4 ± 1.4 cm, 62.4 ± 1.5 kg, 21.5 ± 0.4 kg/m^2^) completed two trials, consisting of two consecutive days (days 1 and 2) of endurance training under low EA (19.9 ± 0.2 kcal/kg fat free mass [FFM]/day, LEA trial) or normal EA (46.4 ± 0.1 kcal/kg FFM/day, NEA trial). The order of these two trials was randomized with at least a 1-week interval between trials. As an endurance training, participants performed 60 min of treadmill running at 70% of maximal oxygen uptake ($\dot{\text{V}}{\text{O}}_{2\text{max}}$) during two consecutive days (on days 1 and 2). On day 1, the endurance training was performed with consumed individually manipulated meals. During the endurance exercise on day 2, exogenous glucose oxidation was evaluated using ^13^C-labeled glucose, and respiratory gas samples were collected. In addition, blood glucose and lactate concentrations were measured immediately after exercise on day 2. Body composition, blood parameters, and resting respiratory gas variables were evaluated under overnight fasting on days 1 and 2. Body weight was significantly reduced in the LEA trial on day2 (day1: 61.8 ± 1.4 kg, day 2: 61.3 ± 1.4 kg, *P* < 0.001). There were no significant differences between trials in ^13^C excretion (*P* = 0.33) and area under the curve during the 60 min of exercise (LEA trial: 40.4 ± 3.1 mmol•60min, NEA trial: 40.4 ± 3.1 mmol•60min, *P* = 0.99). However, the respiratory exchange ratio (RER, LEA trial: 0.88 ± 0.01, NEA trial: 0.90 ± 0.01) and carbohydrate oxidation (LEA trial: 120.1 ± 8.8 g, NEA trial: 136.8 ± 8.6 g) during endurance exercise showed significantly lower values in the LEA trial than in the NEA trial (*P* = 0.01 for RER and carbohydrate oxidation). Serum insulin and total ketone body concentrations were significantly changed after a day of endurance training under low EA (*P* = 0.04 for insulin, *P* < 0.01 for total ketone). In conclusion, low EA during endurance exercise reduced systemic carbohydrate oxidation; however, exogenous glucose oxidation (evaluated by ^13^C excretion) remained unchanged during exercise under low EA.

## Introduction

Acute strenuous exercise suppresses appetite and reduces subsequent energy intake [1–3]. Insufficient energy intake relative to exercise energy expenditure during training or a competitive season is induced among athletes [4–7]. Furthermore, low energy availability (EA) is frequently observed among athletes [8]. EA is defined as the amount of ingested energy remaining for bodily functions and physiological processes [9] and may be more reflective of the increase in energy expenditure during exercise than the energy balance [10]. We previously demonstrated that three days of endurance training under low EA decreases about 30% of muscle glycogen content [11]. In addition, some previous studies demonstrated that low EA impaired exercise performance among swimmers [12] and endurance runners [13]. Moreover, the negative influences of low EA on endocrine response, bone metabolism and psychological health were evident [14–16].

Generally, carbohydrate metabolism during endurance exercise has a critical role in energy production. Among the several negative outcomes of low EA, the influence of low EA on exogenous carbohydrate excretion during endurance exercise remains unclear. Several studies have attempted to clarify the ^13^C glucose excretion kinetics during endurance exercise under reduced muscle glycogen content [17–19]. However, these studies prepared unpractical strategies in the laboratory setting to highlight marked differences in muscle glycogen content at the onset of the ^13^C-test (e.g., comparison of trials without exercise or conducting prolonged exercise to deplete muscle glycogen content prior to the ^13^C-test [17,19]). These procedures do not necessarily mimic the real-life situations among athletes. As previously indicated, low EA frequently occurs, and many athletes perform their training under low EA [5,6]. Therefore, from a realistic viewpoint, it is important to investigate how exogenous glucose is utilized during endurance exercise under low EA.

The purpose of the present study was to determine energy metabolism, especially exogenous carbohydrate metabolism, during endurance exercise under low EA. We hypothesized that exogenous glucose oxidation would be enhanced during endurance exercise under low EA compared with the same exercise under normal EA.

## Materials and methods

### Participants

Ten active males (age [mean ± standard error, SE]: 21.4 ± 0.6 years, height: 170.4 ± 1.4 cm, weight: 62.4 ± 1.5 kg, body mass index: 21.5 ± 0.4 kg/m^2^, maximal oxygen uptake [$\dot{\text{V}}{\text{O}}_{2\text{max}}$]: 56.5 ± 1.3 mL/kg/min) participated in this study. Participants were informed of the purpose, experimental procedure, and potential risk in this study and written informed consent was obtained. This study was approved by the Ethics Committees of Ritsumeikan University (Shiga, Japan).

### Experimental design

Participants conducted two consecutive days of endurance training (days 1 and 2) under low EA (LEA trial) and normal EA (NEA trial) with a crossover design. The washout period was at least 1 week. During each training period, participants were provided individually manipulated meals to adjust each EA.

**Fig 1:**
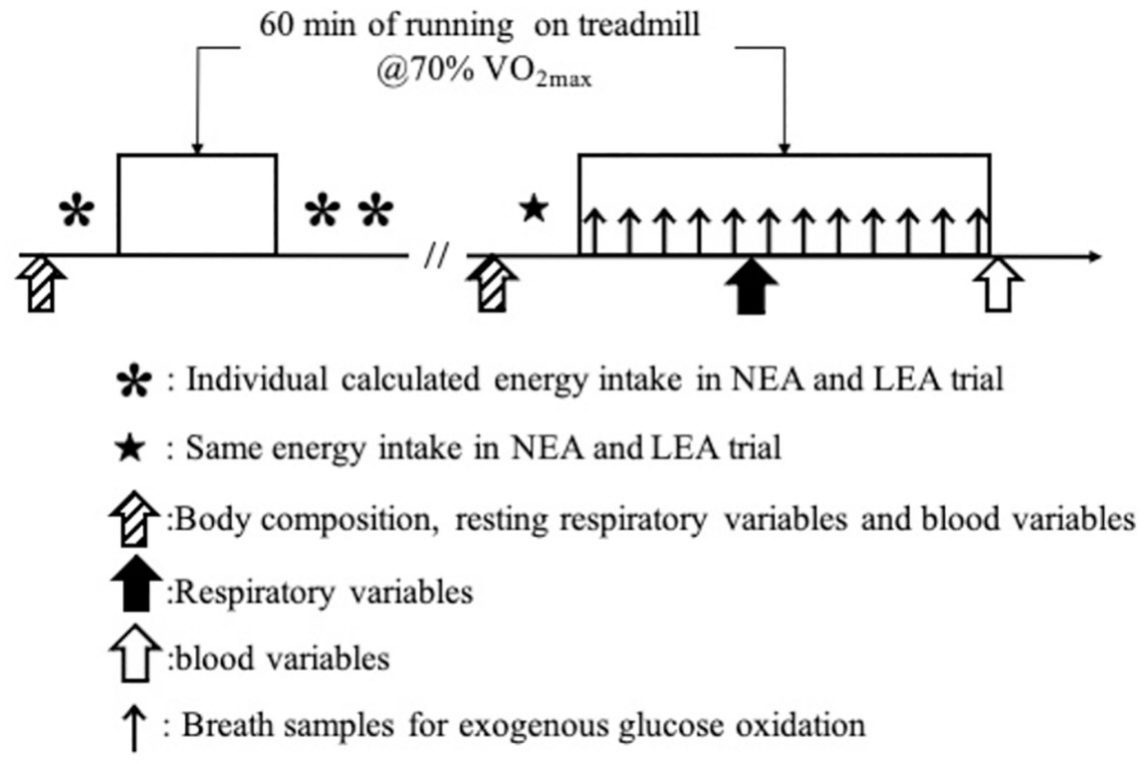
Study design.

Under overnight fasting on days 1 and 2, body composition, blood parameters, and respiratory gas variables were evaluated. On day 1, a single session of endurance training (60 min, 70% of $\dot{\text{V}}{\text{O}}_{2\text{max}}$) was performed. On day 2, participants consumed ^13^C-labeled glucose before starting a single session of endurance training (60 min, 70% of $\dot{\text{V}}{\text{O}}_{2\text{max}}$), and exogenous glucose utilization was subsequently evaluated using the ^13^C-test. Also, respiratory gas samples were collected for 27–30 min during the 60 min of endurance exercise. Blood samples were measured immediately after exercise. All participants stayed in the same accommodation on the day before the experiment (Fig 1).

### Training protocol on days 1 and 2

As endurance training, 60 min of running on a treadmill (Elevation Series E95Ta; Life Fitness, Ltd., Franklin Park, IL, USA) at 70% of $\dot{\text{V}}{\text{O}}_{2\text{max}}$ was performed in the morning. During the exercise, heart rate and rating perceived exertion (RPE) were evaluated continuously and *ad libitum* water intake was recorded.

### Manipulation of EA

EA was determined as daily energy intake minus energy expenditure during exercise relative to free fat mass (FFM) [9]. In the present study, EA was set as 15 kcal/FFM/day in the LEA trial or 45 kcal/FFM/day in the NEA trial. Energy expenditure during exercise was estimated as previously described [11]. Shortly, energy expenditure during 60 min of running at 70% of $\dot{\text{V}}{\text{O}}_{2\text{max}}$ was calculated from $\dot{\text{V}}{\text{O}}_{2}$ and $\dot{\text{V}}{\text{CO}}_{\text{2}}$ using Weir`s equation [20]. The $\dot{\text{V}}{\text{O}}_{2}$ and $\dot{\text{V}}{\text{CO}}_{\text{2}}$ values were estimated from the regression formula between running velocity and respiratory valuables during $\dot{\text{V}}{\text{O}}_{2\text{max}}$ test. Energy intake was calculated from the energy expenditure to meet, individually, each EA.

### Measurements

#### Maximal oxygen uptake ($\dot{\text{V}}{\text{O}}_{2\text{max}}$)

$\dot{\text{V}}{\text{O}}_{2\text{max}}$ was determined by an incremental running test using a treadmill (Valiant; Load B.V., Groningen, The Netherlands) to calculate exercise intensity during the training. The test was started at 10 km/h, and the load was increased every 2 min until 14 km/h. After the velocity reached 14 km/h, it was increased by 0.6 km/h every minute until voluntary exhaustion. During the test, respiratory gas samples were collected to evaluate $\dot{\text{V}}{\text{O}}_{2}$, carbon dioxide output ($\dot{\text{V}}{\text{CO}}_{\text{2}}$), ventilatory volume ($\dot{\text{V}}\text{E}$), and the respiratory exchange ratio (RER) using an automatic gas analyzer (AE 300S; Minato Medical Science Co., Ltd., Osaka, Japan). The values were averaged every 30 s.

#### Body composition

Every morning on days 1 and 2 following overnight fasting (8:00), body weight, skeletal muscle mass, FFM, and body fat were evaluated with the bioimpedance technique using a body composition analyzer (InBody770; InBody Japan Inc., Tokyo, Japan).

#### Respiratory gas samples

Resting respiratory gas samples were collected on days 1 and 2 following overnight fasting (8:10–8:40). Participants sat on comfortable chair followed by sufficient rest (at least 20 min), and respiratory gas samples were measured during sitting for 10 min using an automatic gas analyzer (AE 300S; Minato Medical Science Co., Ltd., Osaka, Japan).

The values were averaged every 30 s. The data during the last 3 min were utilized for further analyses. Also, respiratory gas samples during exercise were evaluated for 27–30 min during 60 min of exercise on day 2 using the same analyzer (AE 300S; Minato Medical Science Co., Ltd., Osaka, Japan). The values were averaged every 30 s. The data were utilized for the last 3 min for further analyses. From the $\dot{\text{V}}{\text{O}}_{2}$ and $\dot{\text{V}}{\text{CO}}_{\text{2}}$, substrate (carbohydrate and fat) oxidation during exercise was estimated using Frayn’s equation [21].

#### Exogenous glucose oxidation (on day 2)

During the training on day 2, ^13^C glucose oxidation was evaluated using the ^13^C-test. Baseline breath samples were collected using a 1.3 L sample bag under overnight fasting (8:00–9:00). In both trials, participants received the same breakfast (9:00) to eliminate the influence of different breakfasts on ^13^C glucose oxidation. Participants consumed 500 mg ^13^C-glucose (D-Glucose-U-^13^C_6_, ^13^C: 99 atom %; Chlorella Industry Co., Ltd., Tokyo, Japan) dissolved in 100 mL purified water followed by 40 min of rest after breakfast. Breath samples were collected every 5 min during the 60 min of exercise (10:00–11:00). The ^13^CO_2_/^12^CO_2_ ratio was determined to express the absolute increase between samplings during exercise and at baseline using an infrared spectrometer (POCone; Otsuka Pharmaceutical Co., Ltd., Tokyo, Japan). ^13^C excretion per unit was calculated as previously described [22–24].

#### Blood parameters

Resting blood samples were collected from the antecubital vein on days 1 and 2 following overnight fasting (8:10–8:40) to evaluate blood glucose, lactate, serum leptin, insulin, total ketone body and free testosterone concentrations. Immediately after exercise on day 2, blood samples were further collected to evaluate blood glucose and lactate concentrations. Serum and plasma samples were obtained by 10 min of centrifugation (3000 rpm) at 4°C and stored at -80°C until analysis. Blood glucose and lactate concentrations were evaluated using a glucose analyzer (Free style; Nipro Co., Osaka, Japan) and a lactate analyzer (Lactate Pro; ARKRAY Co., Kyoto, Japan) immediately after blood collection. Serum leptin concentrations were measured using an enzyme-linked immunosorbent assay (ELISA) kit (R&D Systems Inc., Minneapolis, MN, USA). The intra-assay coefficient of variation (CV) for the ELISA was 1.1%. Serum insulin, total ketone body and free testosterone concentrations were assayed in a clinical laboratory (SRL, Inc., Tokyo, Japan). The intra-assay CVs were 2.2% for insulin, 2.6% for total ketone body concentration, and 7.1% for free testosterone.

### Statistical analyses

Data are expressed as the mean ± SE. Sample size was determined based on the previous study [11], which investigated the effect of low EA on muscle glycogen content. Moreover, considering the effects of reduced muscle glycogen with a day of low EA on metabolic adaptations, effect size (d) was expected to be 1.2 (for comparing the relative changes in muscle glycogen following a day of endurance training under LEA and NEA trial [11]). Therefore, effect size for comparing ^13^C excretion between the two trials was also expected to be 1.2. The results of the power analysis using GPOWER Version 3.1.9 (University of Dusseldorf, Germany) indicated a minimum sample size as eight with power of 0.8 and α level of P ≤ 0.05. With the possibility of dropouts, we recruited ten active males. Time-course changes in energy expenditure, body composition, blood parameters, ^13^C excretion, and respiratory parameters were compared using two-way repeated-measures analysis of variance (ANOVA) to determine the interaction (trial × time) and main effects (trial and time). When ANOVA revealed a significant interaction or main effect, the Tukey-Kramer post-hoc test was performed. Energy intake, energy availability, macronutrient intake during training period and respiratory gas samples during exercise on day 2 were compared between the two trials using the paired *t*-test. Statistical significance was accepted as a p-value < 0.05.

## Results

### Running distance, heart rate, and RPE on days 1 and 2

Average running distance (LEA trial: 11.7 ± 0.3 km/day, NEA trial: 11.8 ± 0.2 km/day) and heart rate (LEA trial: 165 ± 4 bpm, NEA trial: 161 ± 5 bpm) on days 1 and 2 were not significantly different between the trials (*P* > 0.05). Although RPE was significantly increased during exercise (*P* < 0.05), there was no significant difference between the trials on days 1 and 2 (*P* > 0.05).

### Energy expenditure, energy intake, EA, and macronutrient intake

There was no significant difference between the trials in energy expenditure during exercise (LEA trial: day1; 700 ± 27 kcal, day2; 693 ± 26 kcal, NEA trial: day1; 703 ± 27 kcal, 694 ± 26 kcal, interaction: *P* = 0.574, main effect of trial: *P* = 0.105, main effect of time: *P* = 0.160). Table 1 demonstrates energy intake, EA, and macronutrient intake ratio during training period (Table 1). Energy intake was significantly lower in the LEA trial than in the NEA trial (*P* < 0.001). Therefore, EA was significantly lower in the LEA trial compared to the NEA trial (*P* < 0.001). For the macronutrient intake ratio, a significant difference between the two trials was found in protein (*P* < 0.001) and carbohydrate intake (*P* = 0.03), whereas fat intake did not differ between the trials (*P* = 0.29). In addition, there was significant difference between trials in macronutrient intake per body weight in protein, fat carbohydrate intake (*P* < 0.001).

**Table 1:** Energy intake, energy expenditure, energy availability and macronutrient intake during training period.

	LEA	NEA	*P*
Energy intake (kcal)	1788 ± 53‡	3250 ± 67	< 0.001
Energy availability (kcal/kg FFM/day)	19.9 ± 0.3‡	46.6 ± 0.2	< 0.001
Protein intake ratio (%)	21.1 ± 0.2‡	19.1 ± 0.1	< 0.001
Fat intake ratio (%)	27.3 ± 0.2	27.8 ± 0.5	0.287
Carbohydrate intake ratio (%)	51.6 ± 0.4‡	53.1 ± 0.6	0.028
Protein intake (g/BW/day)	1.51 ± 0.02‡	2.49 ± 0.03	< 0.001
Fat intake (g/BW/day)	0.87 ± 0.02‡	1.60 ± 0.03	< 0.001
Carbohydrate intake (g/BW/day)	3.71 ± 0.08‡	6.84 ± 0.15	< 0.001

Values are means ± SE.

^‡^: *P* < 0.05 vs. NEA.

LEA: Low energy availability. NEA: Normal energy availability. BW: Body weight.

### Body composition

Changes in body composition are shown in Table 2. Baseline values did not differ between trials among any parameters. For body weight, two-way ANOVA revealed a significant interaction (trial × time, *P* < 0.001), main effect of the trial (*P* = 0.02), and time (*P* = 0.03). Body weight in the LEA trial was significantly decreased on day 2 with a significantly lower value than that in the NEA trial (*P* < 0.05). For skeletal muscle and FFM, there was no significant interaction (trial × time, *P* = 0.14), main effect for trial (*P* = 0.21) and time (*P* = 0.12). For fat mass, significant interaction (trial × time, *P* = 0.01) and main effect for time (*P* = 0.01) were found. Fat mass was significantly lower in the LEA trial than in the NEA trial (*P* < 0.05).

**Table 2:** Body composition during training period.

		Day1	Day2	Interaction (trial × time)	Main effect	
					Trial	Time
Body weight (kg)	LEA	61.8 ± 1.4	61.3 ± 1.4*‡	< 0.001	0.019	0.029
	NEA	61.9 ± 1.3	62.1 ± 1.3*			
Skeletal muscle (kg)	LEA	51.4 ± 0.9	51.5 ± 0.9	0.142	0.213	0.124
	NEA	51.6 ± 0.9	52.0 ± 0.9			
Fat free mass (kg)	LEA	54.5 ± 0.9	54.6 ± 1.0	0.113	0.198	0.113
	NEA	54.7 ± 0.9	55.1 ± 0.9			
Fat mass (kg)	LEA	7.4 ± 0.5	6.7 ± 0.5*‡	0.007	0.513	0.005
	NEA	7.2 ± 0.6	7.1 ± 0.5			

Values are means ± SE.

*: *P* < 0.05 vs. Day1.

^‡^: *P* < 0.05 vs. NEA.

LEA: Low energy availability. NEA: Normal energy availability.

### ^13^C excretion

**Fig 2:**
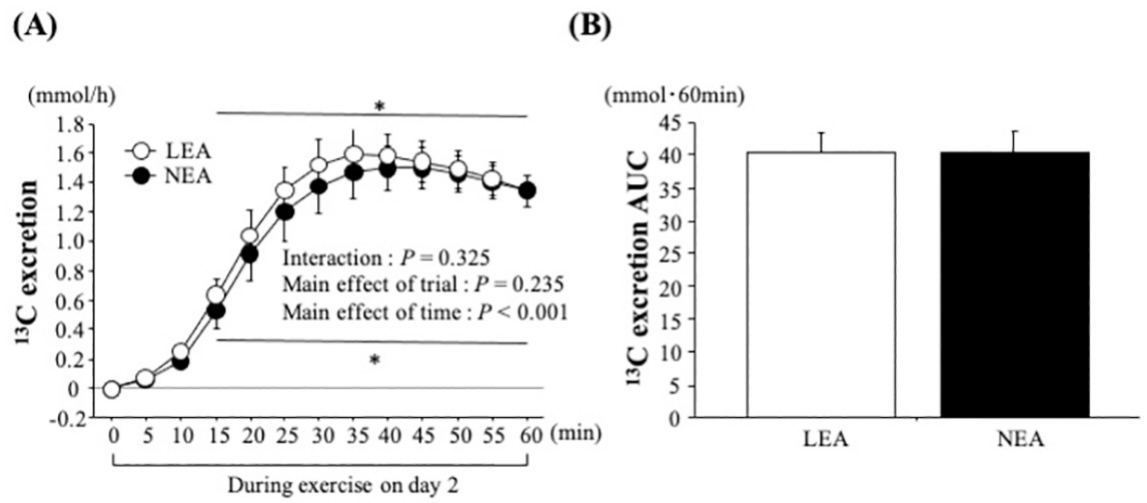
Changes in ^13^C excretion (A) and the AUC for 60 min (B) during exercise on day 2. Values are mean ± SE. *: *P* < 0.05 vs. 0 min.

Fig 2 shows time-course changes in ^13^C excretion and the area under the curve (AUC) for 60 min during exercise (Fig 1). Two-way ANOVA showed a significant main effect of time (*P* = 0.03). Although ^13^C excretion was significantly increased with exercise in both trials (*P* < 0.05), there was no significant difference between trials at any time point (*P* > 0.05). The AUC value during exercise did not differ between trials (*P* = 0.99).

### Blood parameters

**Fig 3:**
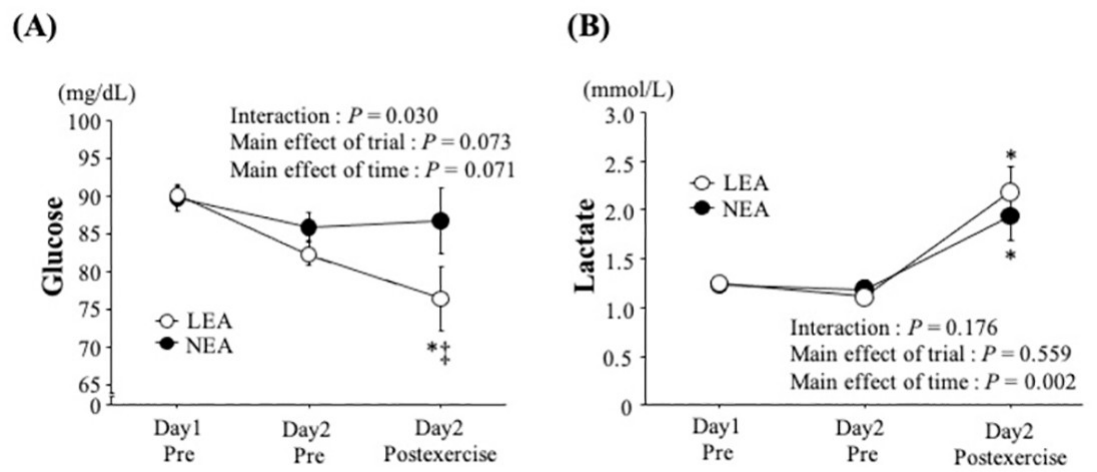
Changes in blood glucose (A) and lactate (B) concentrations. Values are mean ±SE. *: *P* < 0.05 vs. Day 1 Pre. ⁑: *P* < 0.05 vs. NEA.

Fig 3 demonstrates time-course changes in blood glucose and lactate concentration (Fig 2). No significant difference between trials was observed at baseline for both parameters. Two-way ANOVA revealed a significant interaction (trial × time) for glucose (*P* = 0.03) and main effect of time for lactate (*P* < 0.01). Blood glucose concentration in the LEA trial was significantly reduced (*P* < 0.05) with significantly lower values than those in the NEA trial immediately after exercise on day 2 (*P* < 0.05). Blood lactate concentration was significantly increased immediately after exercise on both trials on day 2 (*P* < 0.05).

**Fig 4:**
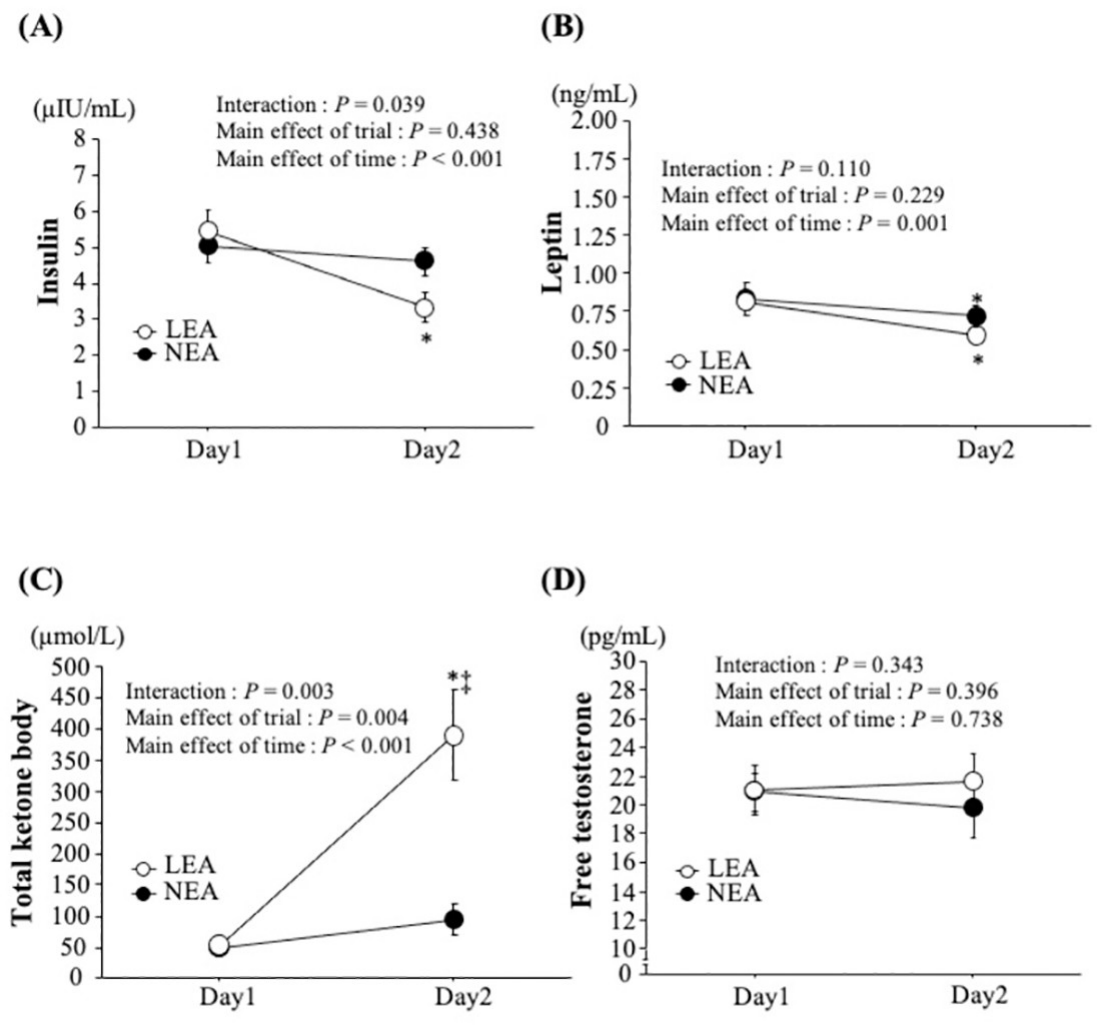
Changes in serum insulin (A), leptin (B), total ketone body (C) and free testosterone (D) concentrations. Values are mean ± SE. *: *P* < 0.05 vs. Day 1. ⁑: *P* < 0.05 vs. NEA.

Time-course changes in serum insulin, leptin, total ketone body and free testosterone concentrations are shown in Fig 4. For any parameter, baseline values were not different between trials on day 1. Two-way ANOVA demonstrated a significant interaction (trial × time, *P* = 0.04) and main effect of time (*P* < 0.001) for serum insulin concentrations. Serum insulin concentrations were significantly decreased in the LEA trial alone (*P* < 0.05). Also, a significant interaction (trial × time, *P* < 0.01) and main effects of trial (*P* < 0.01) and time (*P* < 0.001) for serum total ketone body concentrations were found. In the LEA trial, serum total ketone body concentrations were significantly increased with a significantly higher value than that in the NEA trial (*P* < 0.05). There was a significant main effect of time for serum leptin concentration (*P* < 0.01). Serum leptin concentrations were significantly reduced in both trials (*P* < 0.05), although there was no significant difference between trials (*P* > 0.05). For serum free testosterone concentrations, no significant interaction (trial × time, *P* = 0.34), main effect for time (*P* = 0.74) and trial (*P* = 0.40) were found.

### Respiratory variables

Table 3 shows the results of resting respiratory parameters on days 1 and 2 (Table 3). A significant difference between trials was not observed on day 1 for all parameters except $\dot{\text{V}}{\text{CO}}_{\text{2}}$ and RER. No significant interaction (trial × time), main effect of time and trial were found for $\dot{\text{V}}{\text{O}}_{2}$, $\dot{\text{V}}\text{E}$, and RMR. For $\dot{\text{V}}{\text{CO}}_{\text{2}}$, a significant interaction (trial × time, *P* = 0.01) and main effect for time (*P* = 0.05) were observed. On day 1, $\dot{\text{V}}{\text{CO}}_{\text{2}}$ was significantly higher in the LEA trial than in the NEA trial (*P* < 0.05). A significant interaction (trial × time, *P* = 0.03) and main effect of time (*P* < 0.01) were observed for RER. On day 1, there was a significant difference between trials (*P* < 0.05). Also, RER in the LEA trial was significantly reduced with a significantly lower value than that in the NEA trial on day 2 (*P* < 0.05).

**Table 3:** Resting respiratory variables and RMR during training period.

		Day1	Day2	Interaction (trial×time)	Main effect	
					Trial	Time
$\dot{\text{V}}{\text{O}}_{2}$ (ml/min)	LEA	222.0 ± 6.4	227.0 ± 6.4	0.897	0.106	0.312
	NEA	215.4 ± 8.0	221.6 ± 6.1			
$\dot{\text{V}}{\text{CO}}_{\text{2}}$ (ml/min)	LEA	186.1 ± 5.5‡	175.5 ±4.8	0.011	0.046	0.66
	NEA	171.2 ± 7.0	177.8 ± 3.8			
RER	LEA	0.84 ± 0.01‡	0.77 ± 0.02*‡	0.027	0.408	0.003
	NEA	0.80 ± 0.02	0.81 ± 0.01			
$\dot{\text{V}}\text{E}$ (L/min)	LEA	8.0 ± 0.3	7.5 ± 0.3	0.167	0.153	0.282
	NEA	7.6 ± 0.3	7.5 ± 0.2			
RMR (kcal/min)	LEA	1.06 ± 0.03	1.07 ± 0.03	0.574	0.105	0.445
	NEA	1.02 ± 0.04	1.05 ± 0.03			

Values are means ± SE.

*: *P* < 0.05 vs. Day1.

^‡^: *P* < 0.05 vs. NEA.

LEA: Low energy availability. NEA: Normal energy availability. $\dot{\text{V}}{\text{O}}_{2}$: Maximal oxygen uptake. $\dot{\text{V}}{\text{CO}}_{\text{2}}$: Carbon dioxide output. $\dot{\text{V}}\text{E}$: Ventilatory volume. RER: Respiratory exchange ratio. RMR: Resting metabolic rate.

Table 4 shows the respiratory samples during exercise on day 2 (Table 4). RER and carbohydrate oxidation were significantly lower in the LEA trial than in the NEA trial (*P* = 0.01 for RER and carbohydrate oxidation). In addition, the LEA trial showed significantly higher fat oxidation (*P* = 0.01).

**Table 4:** Respiratory variables during exercise on day 2.

	LEA	NEA	*P*
_$\dot{\text{V}}{\text{O}}_{2}$_ (ml/min)	2524.0 ± 126.0	2525.8 ± 120.3	1.00
_$\dot{\text{V}}{\text{CO}}_{\text{2}}$_ (ml/min)	2220.6 ± 112.1	2283.0 ± 109.3	0.13
RER	0.88 ± 0.01‡	0.90 ± 0.01	0.01
$\dot{\text{V}}\text{E}$ (L/min)	67.2 ± 3.3	68.3 ±2.9	0.56
CHO oxidation (g)	120.1 ± 8.8‡	136.8 ± 8.6	0.01
Fat oxidation (g)	30.4 ± 3.0‡	24.3 ± 2.6	0.01

Values are means ± SE.

^‡^: *P* < 0.05 vs. NEA.

LEA: Low energy availability. NEA: Normal energy availability. $\dot{\text{V}}{\text{O}}_{2}$: Maximal oxygen uptake. $\dot{\text{V}}{\text{CO}}_{\text{2}}$: Carbon dioxide output. $\dot{\text{V}}\text{E}$: Ventilatory volume. RER: Respiratory exchange ratio. CHO oxidation: Carbohydrate oxidation.

## Discussion

The present study investigated the effects of low EA induced by lowered energy intake on carbohydrate metabolism during a single session of endurance exercise. The main finding of the study was that systemic carbohydrate oxidation (evaluated by RER and fat oxidation) was reduced during endurance exercise with low EA, whereas exogenous glucose oxidation (evaluated by ^13^C excretion) was not affected.

In this study, evaluation of exogenous glucose excretion using ^13^C was investigated during exercise in addition to systemic metabolic variables ($\dot{\text{V}}{\text{O}}_{2}$, $\dot{\text{V}}{\text{CO}}_{\text{2}}$, and RER) and blood parameters. We hypothesized that exogenous glucose oxidation would be greater to compensate for lowered endogenous (muscle and liver) glycogen content in the LEA trial. However, endurance exercise under low EA did not affect exogenous carbohydrate oxidation, whereas systemic carbohydrate metabolism was attenuated (e.g., increased fat oxidation and decreased RER). Several studies have investigated the effect of different muscle glycogen contents on exogenous and endogenous glucose oxidation during exercise [17–19,25]. Most previous studies restricted carbohydrate intake (carbohydrate availability) and/or utilized glycogen-depleted exercise to achieve apparent differences in muscle glycogen content at the onset of the exercise, which may not mimic real situations in the field. To the best of our knowledge, the present study was the first to investigate ^13^C excretion during endurance exercise under manipulated EA without profound reduced carbohydrate availability and/or glycogen-depleted exercise. Although we did not determine muscle and liver glycogen contents, it was expected that muscle glycogen content was lowered about 20% at onset of exercise on day 2 [11]. In addition, serum ketone body concentrations were significantly increased in LEA trial, indicating that hepatic fat metabolism was enhanced, which suggests lowered liver glycogen content. Furthermore, it was notable to find that blood glucose concentration was significantly decreased in the LEA trial alone, immediately after exercise. Hespel et al. [26] reported that glucose uptake is facilitated during exercise under low muscle glycogen content. Taken together, it is possible that ^13^C glucose uptake is facilitated to resynthesize glycogen content within muscle and liver, and not for utilization as an energy source during exercise [27]. In accordance with the present study, Margolis et al. [25] reported that endurance exercise with lowered muscle glycogen content (~50% of reduction) elevates whole-body fat oxidation whereas exogenous carbohydrate oxidation did not alter. However, the authors suggested that fat was utilized as a fuel primarily when exercising with low muscle glycogen, not increased glucose uptake. On the other hand, the study utilized severe restriction of carbohydrate availability (1.5 g/BW/day) without lowering energy intake (i.e., not altering EA) to manipulate muscle glycogen content [25], indicating that the effects of high fat intake should be considered. Furthermore, in the present study, enhanced gastric emptying might affect exogenous glucose oxidation kinetics during exercise only because of low EA due to a negative energy balance, but not low carbohydrate availability. As a support of this idea, ghrelin (an orexigenic hormone), which is increased under fasting and lowered energy intake [28], enhances gastric emptying [29].

EA was manipulated as 15 kcal/FFM/day in the LEA trial or 45 kcal/FFM/day in the NEA trial. However, serum leptin and free testosterone concentrations, as indications of the energy balance [30,31], did not show significant changes in the LEA trial, which was not consistent with previous studies [11,32]. Because dietary manipulation using low EA lasted only a single day, it may be insufficient to alter energy balance-associated endocrine regulation. However, serum ketone body concentration (an indication of hepatic fat metabolism) was markedly increased in the LEA trial on day 2. In addition, serum insulin concentration was significantly decreased in the LEA trial alone. These alterations indicate that energy metabolism was modified (e.g., augmented fat metabolism) during the two consecutive days of endurance training with low EA, despite the lack of differences in leptin and free testosterone.

There are several limitations in the present study. First, it still remains unclear where the exogenous glucose was oxidated mainly during endurance exercise. It is speculated that ^13^C excretion affects exogenous glucose oxidation within liver rather than muscles. Because contributions of exogenous and endogenous glucose oxidation within muscles and liver during exercise were complicated, further evidences (e.g., muscle and liver glycogen utilization during exercise, blood glucose disappearance) are required. In addition, the present study did not evaluate participant’s daily EA, especially, before this experiment and during wash-out period. Since daily nutrient intake and energy expenditure during exercise play important roles in energy metabolism, it might be necessary to measure these parameters. However, the present study prepared at least a week (up to two weeks) of wash-out periods. Thus, such short period is unlikely to change these parameters because these parameters are susceptible to environment factors (e.g., seasonal differences), suggesting that EA was thought to be robust throughout the whole experimental period.

## Conclusion

Endurance exercise under low EA significantly decreased systemic carbohydrate oxidation during exercise compared to the same exercise under normal EA. However, it did not affect exogenous glucose oxidation. The present results are possible to contribute to developing nutrient strategies regarding potent supplementations during prolonged exercise under low EA among athletes. Although the present study evaluated the influence of a single day of endurance training under low EA on carbohydrate metabolism, how carbohydrate metabolism is changed during exercise under sustained low EA should be investigated.
